# Predicting respiratory decompensation in mechanically ventilated adult ICU patients

**DOI:** 10.3389/fphys.2023.1125991

**Published:** 2023-04-14

**Authors:** Yvette Tan, Michael Young, Akanksha Girish, Beini Hu, Zina Kurian, Joseph L. Greenstein, Han Kim, Raimond L Winslow, James Fackler, Jules Bergmann

**Affiliations:** ^1^ Whiting School of Engineering, Johns Hopkins University, Baltimore, MD, United States; ^2^ Institute for Computational Medicine, Johns Hopkins University, Baltimore, MD, United States; ^3^ Department of Anesthesiology and Critical Care Medicine, School of Medicine, Johns Hopkins University, Baltimore, MD, United States

**Keywords:** mechanical ventilalion, ventilator-associated complications, ventilator-associated event (VAE), random forest, respiratory decompensation, machine learning—ML

## Abstract

**Introduction:** Mechanical ventilation is a life-saving treatment in the Intensive Care Unit (ICU), but often causes patients to be at risk of further respiratory complication. We created a statistical model utilizing electronic health record and physiologic vitals data to predict the Center for Disease Control and Prevention (CDC) defined Ventilator Associated Complications (VACs). Further, we evaluated the effect of data temporal resolution and feature generation method choice on the accuracy of such a constructed model.

**Methods:** We constructed a random forest model to predict occurrence of VACs using health records and chart events from adult patients in the Medical Information Mart for Intensive Care III (MIMIC-III) database. We trained the machine learning models on two patient populations of 1921 and 464 based on low and high frequency data availability. Model features were generated using both basic statistical summaries and tsfresh, a python library that generates a large number of derived time-series features. Classification to determine whether a patient will experience VAC one hour after 35 h of ventilation was performed using a random forest classifier. Two different sample spaces conditioned on five varying feature extraction techniques were evaluated to identify the most optimal selection of features resulting in the best VAC discrimination. Each dataset was assessed using K-folds cross-validation (*k* = 10), giving average area under the receiver operating characteristic curves (AUROCs) and accuracies.

**Results:** After feature selection, hyperparameter tuning, and feature extraction, the best performing model used automatically generated features on high frequency data and achieved an average AUROC of 0.83 ± 0.11 and an average accuracy of 0.69 ± 0.10.

**Discussion:** Results show the potential viability of predicting VACs using machine learning, and indicate that higher-resolution data and the larger feature set generated by tsfresh yield better AUROCs compared to lower-resolution data and manual statistical features.

## 1 Introduction

Mechanical ventilation is a life-saving treatment for patients with respiratory failure. Every year in the United States, up to 800,000 patients receive mechanical ventilation treatment (Cocoros et al., 2016). However, ventilated patients are at risk of further respiratory decompensation that increases the longer a patient is mechanically ventilated (Magill et al., 2013). Decompensation causes include disease progression (e.g., infection spreading to lungs or within lungs) and therapy complications (e.g., fluid overload from volume resuscitation, traumatic injury from ventilator pressures). Identifying patients at risk for further decompensation can aid clinical decision making for mechanically ventilated patients. In this paper, we present a predictive, early-warning classifier model for respiratory decompensation of mechanically ventilated patients.

Training predictive models and evaluating their performance requires meaningful labels of clinical events (Rajkomar et al., 2019). Our respiratory decompensation label is based on the ventilator associated complication (VAC) event from the ventilator associated events (VAE) surveillance framework introduced by Magill et al. (2013). Designed as an objective replacement for the ventilatory associated pneumonia (VAP) surveillance definition, VAEs include infectious and non-infectious causes of respiratory decompensation and have demonstrated association with patient outcomes including mortality and length of stay (Klompas, 2019). A VAC is a period of 48 h of stable/improving ventilator settings, followed by 48 h of increased ventilator settings. To create clinically relevant labels, we use the midpoint of the VAC window, when the patient transitions from stable/improving ventilator settings to increased settings, as the time for respiratory decompensation to train and evaluate our models. We also label VACs on an hourly basis (versus the daily basis used in surveillance).

To predict of VAC risk, our models use features derived from patient demographics, EHR data, and physiologic time series data.

## 2 Materials and methods

Our study aims were twofold:

1. Build a model to predict VACs 1 h before occurrence using 35 h of ventilation data
2. Evaluate different feature extraction techniques on our model

In this study, we wanted to compare different feature extraction techniques on different granularities of data. We looked at manual statistical features vs. automated feature extraction, and high-resolution vs. low-resolution data.

To create our models, we leveraged data from MIMIC-III, an open-access dataset (Johnson et al., 2016), and utilized machine learning techniques to construct various models. Our results have the potential to inform clinicians on the need for timely critical interventions during the ICU stay of mechanically ventilated patients before obvious signs of decompensation. With a clinically significant early warning system, the monitoring physician will have a risk score, in addition to other physiological measurements, as evidence to make changes to treatment protocol if necessary. Earlier intervention on the part of the clinician can help mitigate and altogether prevent complications that have been predicted to occur. The prevention of a VAC improves patient outcomes by shortening duration of ICU stay, reducing hospital costs, and preventing further mechanical injury (Dasta et al., 2005).

### 2.1 Prior work

Prior work in this area includes algorithms applied to other ICU classifier events such as unplanned intubation in Trauma ICU patients (Schmidt et al., 2014), moving of patients to the ICU within 12 h (Gursel and Demirtas, 2006; Blackburn et al., 2017), as well as the likelihood of, mortality due to, or survival despite events similar to VACs such as sepsis (Calvert et al., 2016; Harrison et al., 2016; Scherpf et al., 2019; Fang et al., 2020), acute respiratory distress syndrome (ARDS), and respiratory failure after initiation of extracorporeal membrane oxygenation (ECMO) treatment (Schmidt et al., 2014). Such studies have produced results with a wide range of AUCs ranging between 0.625 and 0.92. A study by Huber et al. (2020) compared various ARDS definitions and associated information during intubation and had a range of AUC scores from 0.620 to 0.824 depending on features used for prediction. Though studies predicting VACs are few, there has been work over related predictive analyses. For example, the APACHE II score has been used to predict mortality from VAP with an AUC of .81 (Schmidt et al., 2014) and VAE definition conditions have been shown to predict poorer outcomes in sepsis patients using a log-rank test (Fang et al., 2020).

Most hospital admission centers calculate critical patient scores within the first 24 h of ICU admission (Gursel and Demirtas, 2006). There is a clear need for a prediction model regarding events prior to patient decompensation, specific to mechanical ventilation (Fang et al., 2020). Other predictive models, such as the likelihood of sepsis, use a similar approach (Calvert et al., 2016; Harrison et al., 2016; Scherpf et al., 2019; Fang et al., 2020). In such models, a heightened sensitivity is of emphasized importance to minimize poor outcomes from false negatives. Prior research indicates both a need for and the potential to create a predictive model that meets these specifications.

### 2.2 Hypotheses

For both cohorts, we built random forest models using manual as well as tsfresh low-frequency features. For the high frequency cohort, we also built a random forest model using the top tsfresh high-frequency features. The main variables we were interested in comparing were cohort size, temporal resolution of data, and automated features. In examining model performance while varying these different aspects, we hypothesized that:

1. Increasing temporal resolution through use of waveform data will improve a random forest model’s ability to predict VACs
2. Increasing the sample size of the training set will improve a random forest model’s ability to predict VACs
3. Use of tsfresh in generating features will improve a random forest model’s ability to predict VACs relative to a model with manually-derived features

### 2.3 Study populations

**FIGURE 1:**
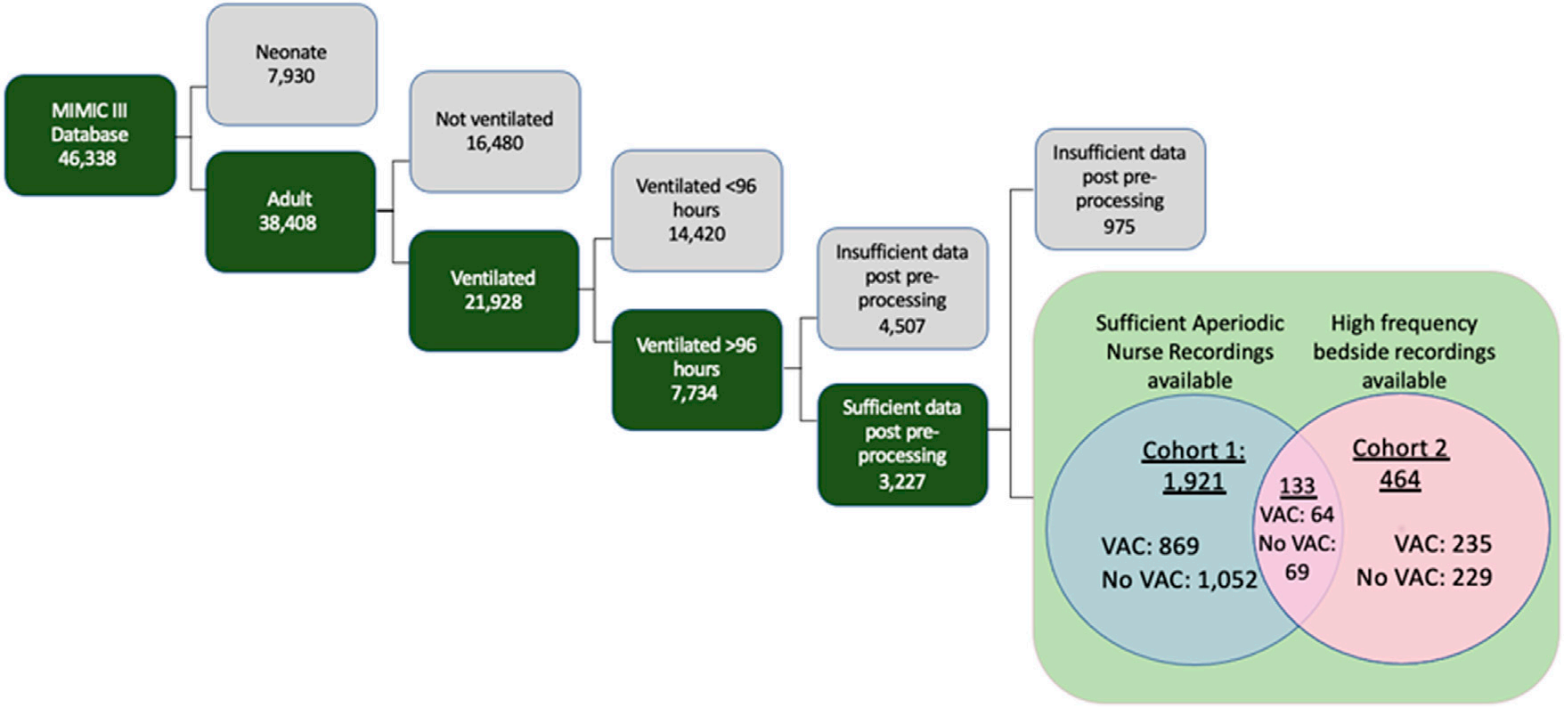
Inclusion criteria for MIMIC-III database to arrive at anticipated patient Cohorts One and Two, both of which were composed of patients who had enough data points for automatic statistical feature extraction methods. Cohort One consists of patients with aperiodic data recorded by nurses and clinicians, and Cohort Two consists of patients with high frequency waveform data.

We selected patients retrospectively from the MIMIC-III Clinical Database. Published by the Massachusetts Institute of Technology (MIT) and Physionet, MIMIC-III contains 46,338 adult and pediatric patients admitted to the critical care units of Beth Isreal Deaconess Medical Center in Boston, Massachusetts between 2001 and 2012. Adult patients were included if they received mechanical ventilation for at least 96 h—the minimum time to be formally at risk for a VAC. Only one ICU stay was considered per patient. Patients with more than 40% of missing low-frequency data were excluded (Figure 1). In addition, we used the MIMIC-III Waveform Database for high-resolution physiologic data. A companion to the original MIMIC-III, this database supplies patient records with automatically collected vital signs at a high temporal resolution, along with numerical records similar to that of MIMIC-III Clinical (Moody et al., 2017). Patients with sufficient data for statistical feature generation were placed in Cohort One. Patients with sufficient physiologic time series data were placed in Cohort Two.

### 2.4 Prediction task

We considered the outcome of VAC, as defined by the CDC to be 48 h of stable or decreasing daily minimum FiO_2_ and PEEP settings, followed by 48 h where either the daily minimum FiO_2_ or PEEP increased by 0.2 or 3 mm Hg respectively. The time of transition from stable/decreasing settings to increasing settings was considered the VAC onset time. For patients at risk of a VAC (having been ventilated for at least 48 h), we then considered the binary prediction at hour of mechanical ventilation with hours labeled as 1 for patients in which a VAC onset occurred and 0 otherwise. Models considered patient demographic variables and patient data from the previous 35 h (chosen *a priori*) to 1 h before VAC onset to make a prediction.

### 2.5 Data preprocessing and missingness strategy

For each patient, demographics (age, gender), labs (arterial pH, PaO_2_, glucose, WBC), nurse recorded vital signs (temperature, heart rate, blood pressure, respiratory rate, SpO_2_), nurse recorded ventilator settings/measurements (PEEP, mean airway pressure), and physiologic time series data were extracted. Physiologically implausible values were removed (Table 1).

**TABLE 1:** Valid physiological vital signs parameter ranges to determine outliers.

	HR (bpm)	RR (breaths/min)	Diastolic B.P. (mmHg)	Systolic B.P. (mmHg)	SpO_2_ (%)	PEEP (cm H_2_0)	FiO_2_ (%)
Lower bound	30	6	20	50	80	0	21
Upper bound	200	50	120	220	100	20	100

Vital signals include heart rate (HR), respiratory rate (RR), diastolic and systolic blood pressure (B.P.), oxygen saturation (SpO_2_), positive end-expiratory pressure (PEEP), and fraction of inspired oxygen (FiO_2_).

Low-frequency values missing for more than 1 h were replaced with the mean value from the training set. High-frequency data were partitioned into 5-min intervals for median down-sampling. After down-sampling, data gaps longer than 1 h (12 consecutive missing values) were filled by linear interpolation in a manner that ensured causality. Data gaps shorter than 1 h were carried forward from the most recent non-null value. For leading null values in the window that could not be replaced using carry-forward (all prior values before the window were also null), the value was backward-filled with the first future non-missing value.

### 2.6 Feature extraction and selection

Different time frames of data before VAC onset were tested for model input with preliminary models, with the best results found using the data 36 h before VAC onset. Data was therefore extracted 36 h before VAC onset, and features were extracted during the first 35 h of this data, allowing for prediction 1 h before VAC onset. Variables with 40% or more missing samples across patients were removed. We computed statistical summaries (mean, variance, number of features, min, max, and range) of 12 h windows and of 3 h windows of all variables, resulting in 278 features. We also used the tsfresh package (Christ et al., 2018) to automatically calculate derived static features from the underlying time-series data, resulting in 916 features from 20 low frequency signals and 12,853 features from 6 high frequency signals.

Highly correlated features were removed when Pearson correlations were greater than 0.95. Forward selection, a stepwise regression technique utilizing significance (*p*-value) levels from model performance, was used to select the most relevant features from each type of data, resulting in a top 35 tsfresh features and 29 manual features from the low frequency data, as well as a top 167 tsfresh features from the high frequency data.

### 2.7 Model training and validation

We trained and compared five different models using random forest classification, as implemented by the scikit-learn library (Pedregosa et al., 2011) in Python 3. For Cohort One, two models were created: one using tsfresh features derived from low-frequency nurse recorded data, and one using manually defined features from the same. For Cohort Two with high frequency data availability, three models were created: one using tsfresh features derived from low-frequency data, one using tsfresh features derived from high-frequency data, and one using manual features from low-frequency data.

The models constructed are summarized below:

1. Tsfresh features derived from low frequency data of 1921 Cohort One patients
2. Tsfresh features derived from low frequency data of 464 Cohort Two patients
3. Tsfresh features derived from high frequency data of 464 Cohort Two patient
4. Manual features derived from low frequency data of 1921 Cohort One patients
5. Manual features derived from low frequency data of 464 Cohort Two patients

Random forests have 4 hyperparameters—the number of trees, the maximum features, the maximum tree depth, and splitting criterion. A hyperparameter search was conducted with the GridSearchCV function from scikit-learn. Model performance was evaluated using 10-fold cross validation (e.g., for hyperparameter setting 10 different models were built using 90% of the data and performance was evaluated on the remaining 10% of the data, then averaged). Model performance was measured by the AUROC. The best performing hyperparameters were then reported as is. Random forest, our choice of classification, is a machine learning algorithm that classifies testing data based on uncorrelated decision trees (set at 512 trees), each of which intuitively ask a sequence of questions about the data until it arrives at a classification. A hyperparameter search gave optimal parameters of “auto” for max features, 500 estimators, a max depth of 9, and the Gini criterion for determining split quality in fitting. However, these settings did not cause model performance to materially deviate from default settings. Random forest models were constructed using features chosen after feature reduction and selective feature elimination. Model outcome was incidence of a VAC during the patient’s ICU stay. Classification results were obtained from K-folds cross-validation (*k* = 10), and summary statistics from the average area under the receiver operating characteristic (AUROC) curves are reported in Table 2.

**TABLE 2:** Summary of model results.

Model	Number of patients	Number of features	AUROC mean ± variance	Accuracy mean ± variance	AUPRC mean ± variance	PPV mean ± variance
M1 EVENTs tsfresh features^a^	1921 (Cohort 1)	35	.7620 ± .0017	.6976 ± .0012	.7359 ± .0025	.6990 ± .0027
M2 EVENTs tsfresh features^b^	464 (Cohort 2)	35	.6262 ± .0069	.5901 ± .0066	.6250 ± .0092	.6061 ± .0084
M3 PTS data tsfresh features^c^	464 (Cohort 2)	167	.8257 ± .0055	.7650 ± .0075	.8261 ± .0070	.7610 ± .0081
M4 Manual features^d^	1921 (Cohort 1)	29	.7916 ± .0010	.7314 ± .0011	.7671 ± .0010	.7266 ± .0034
M5 Manual features^e^	464 (Cohort 2)	29	.7373 ± .0023	.6811 ± .0019	.7091 ± .0075	.6770 ± .0027

Number of patients indicates the cohort used for both training and testing of models. The number of features column represents the number of features found to give the largest AUC and was found using the forward selection technique for feature selection.

^a^ Low-resolution features generated using tsfresh on Cohort 1.

^b^ Low-resolution features generated using tsfresh on Cohort 2.

^c^ High-resolution features generated using tsfresh on Cohort 2.

^d^ Low-resolution features generated manually on Cohort 1.

^e^ Low-resolution features generated manually on Cohort 2.

## 3 Results

Cohort One, patients with sufficient EHR data for statistical feature generation, included 1921 patients experiencing 869 VACs. Cohort Two, patients with sufficient physiologic time series data for statistical feature generation, included 464 patients experiencing 235 VACs. (133 patients were in both cohorts). Table 3 describes the characteristics of these cohorts.

**TABLE 3:** VAC Cohort patients.

	Cohort 1	Cohort 2
Unique Subjects (n)	1921	464
Age, years [mean, (s.d.)]	69.3, (43.49%)	66.3, (36.6%)
Male gender [n, (%)]	1,126, (58.7%)	272, (58.6%)
Ventilator Duration (mean hours)	236	206
Length of ICU stay (mean days)	13.84	12.03
Hospital mortality [n, (%)]	637, (33.2%)	154, (33.2%)
Patients with VAC	869	235

### 3.1 Feature generation and selection

Tsfresh generated 916 statistical features from EHR data in cohort one. 350 features with Pearson correlation greater than 0.95 were removed. 531 additional features were removed by forward selection, resulting in 35 tsfresh features on low-resolution physiologic data. On cohort two, tsfresh generated 12,853 statistical features from PTS data, of which 12,491 were removed by Pearson correlation and 195 were removed by forward selection, resulting in 167 tsfresh features on high-resolution physiologic data.

### 3.2 Model performance

**FIGURE 2:**
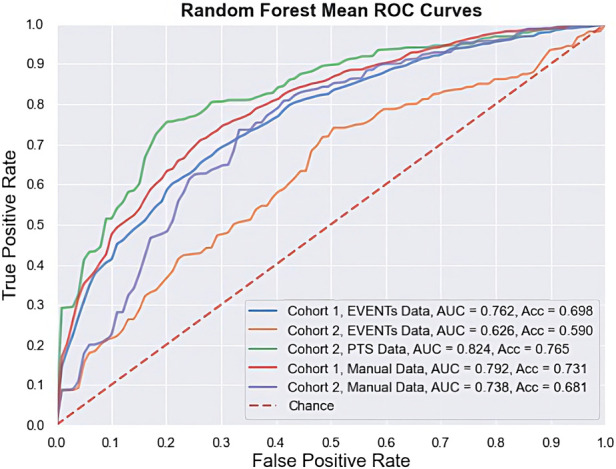
Plot of random forest mean AUCs.

Table 2 and Figure 2 summarize model performance (AUROC and accuracy) of the five models using 10-fold cross validation. On Cohort One, automated low-resolution features (M1) achieved an AUC of 0.762 ± 0.002 and manual features (M4) achieved an AUROC of 0.792 ± 0.001. On cohort two, automated high-resolution features (M3) achieved an AUROC of 0.826 ± 0.006, automated low-resolution features (M2) achieved an AUROC of 0.626 ± 0.007, and manual features (M5) achieved an AUROC of 0.737 ± 0.002.

### 3.3 Feature importance

These most relevant features constitute a set of possible baseline risk factors, and derive from the signals of airway pressure, SpO_2_, respiratory rate (RR), diastolic blood pressure (DBP), systolic blood pressure (SBP), heart rate (HR), PaO_2_, glucose stick levels, mean arterial pressure (MAP), Glasgow coma scale score (GSC score), temperature (TEMP), and white blood cell count (WBC). Additional static features included a binary categorical variable for if a neuromuscular blocker was prescribed, shock index (HR/SBP), age, gender, time since admission to the ICU, and the number of vital sign measurements recorded in a patient’s data collection window.

## 4 Discussion

The CDC ventilator associated complication (VAC) surveillance criteria identifies a range of complications associated with mechanical ventilation in adult patients (Muscedere et al., 2013) and has been associated with increased morbidity/mortality (Muscedere et al., 2013).

Clinically, model outputs can be applied in at least three ways. First, when an individual patient’s score crosses a threshold associated with high specificity, immediate diagnostics and interventions could be triggered to assess for early signs of complications (such as pneumothorax) and optimize pulmonary function (such as more aggressive pulmonary toilet). Second, an individual patient’s score trajectory over time may provide early warning about occult developments that may progress into decompensation. Finally, relative scores in a cohort of patients, such as ICU provider’s panel, could help triage both attention and resources to patients at greatest risk of decompensation.

Methodologically, we demonstrate the viability of automated feature generation from high-resolution physiologic time series data. Packages like tsfresh can generate a large number of non-linear summarizations of physiologic time series data, some more related to the outcome than manually constructed features. After removal of a) features highly correlated with other features and b) features poorly correlated with the outcome while controlling for other features, model performance (AUROC 0.826 for M3) exceeds that of manual features (AUROC 0.737 for M5). This result did not hold for automated features generated from low resolution data, where performance was consistently lower than for models built with manual features (M1 vs. M4 and M2 vs. M5) and lower than for models built with automated features from high-resolution. We also demonstrate, not unexpectedly, that models with identical features but trained on more patients have better performance (M1 vs. M2 and M4 vs. M5).

**FIGURE 3:**
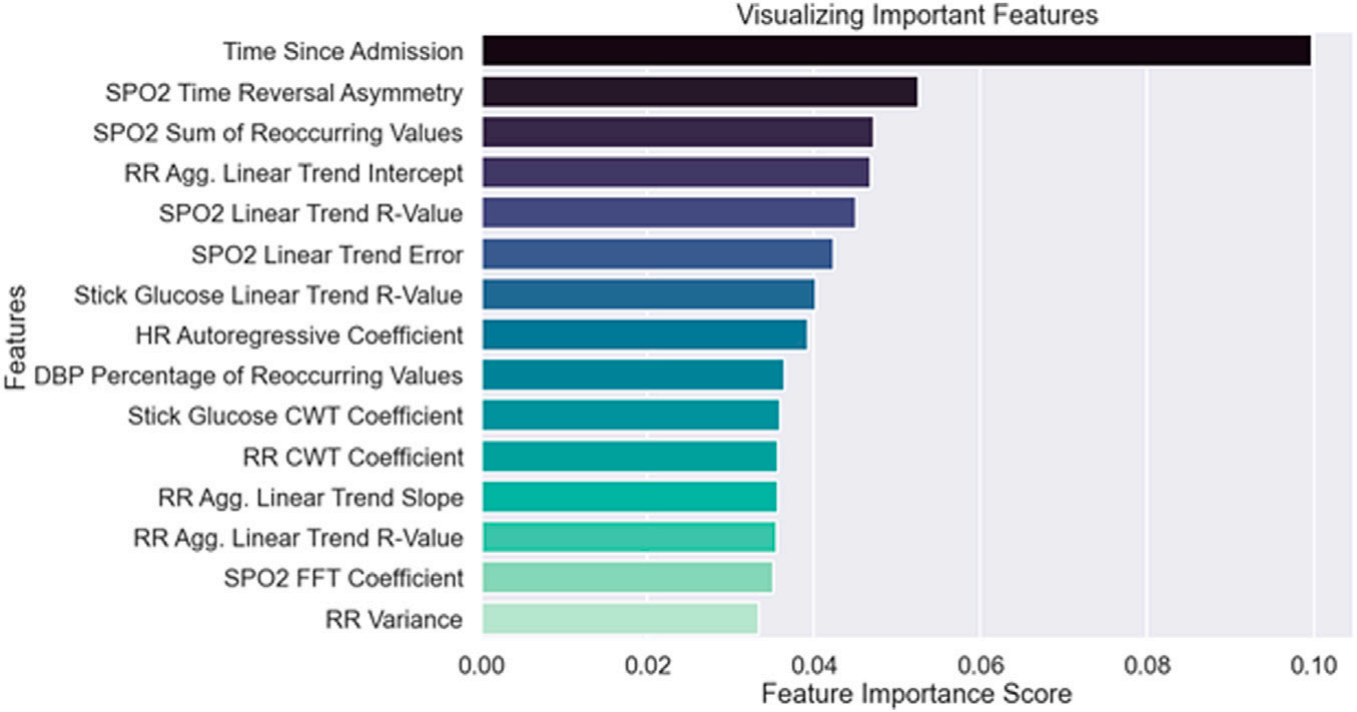
Top 15 significant Features from Cohort 1 tsfresh EVENTs features (M1).

**FIGURE 4:**
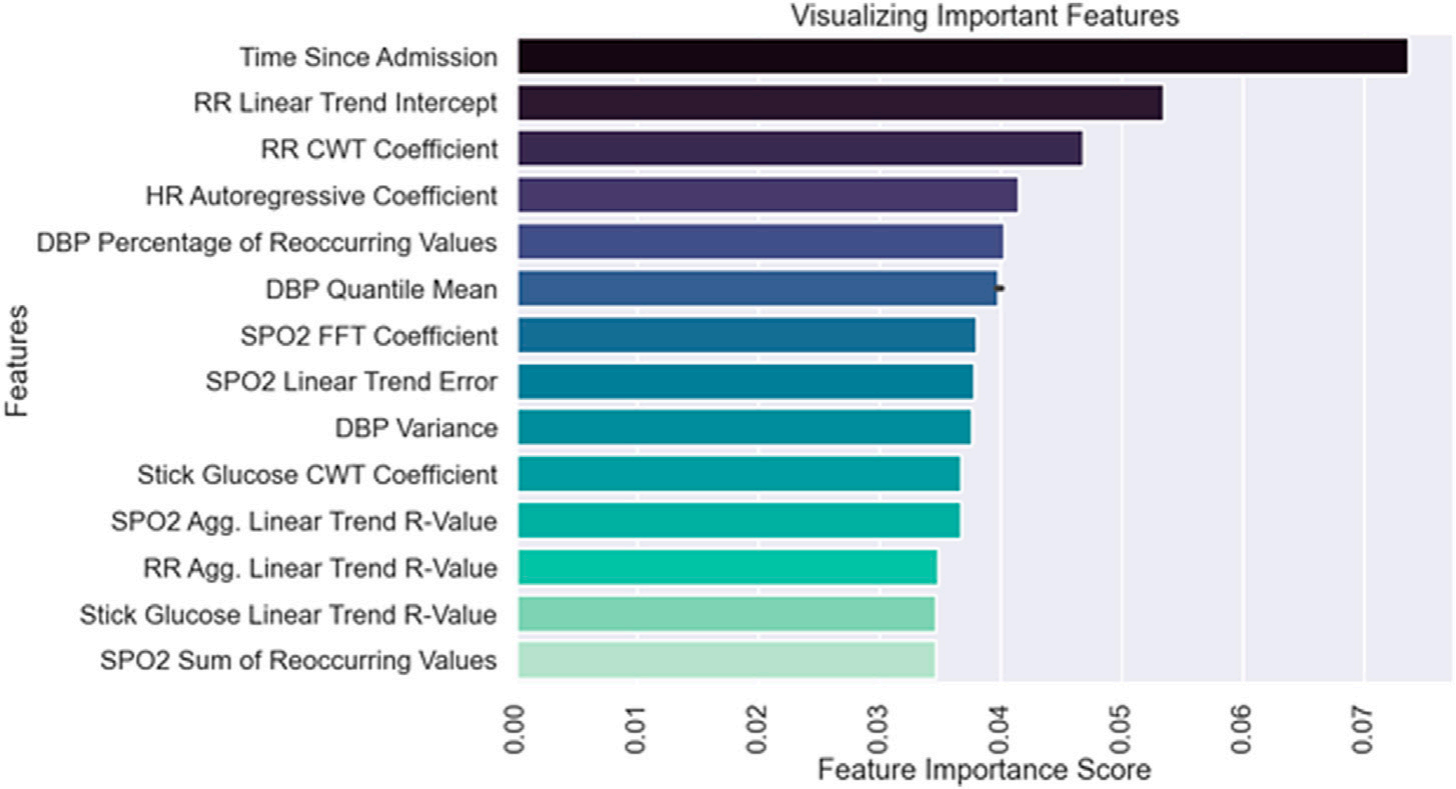
Top 15 significant Features from Cohort 2 tsfresh EVENTs features (M2).

**FIGURE 5:**
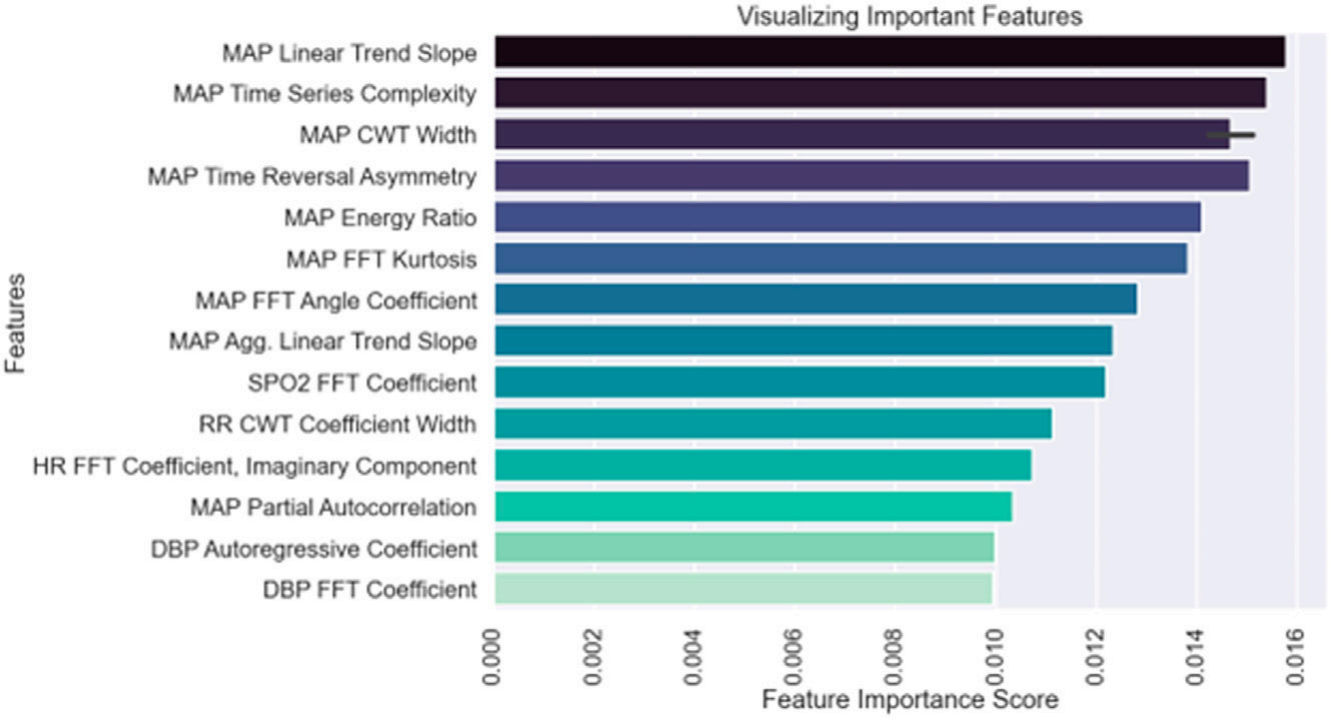
Top 15 significant features from PTS data (M3).

**FIGURE 6:**
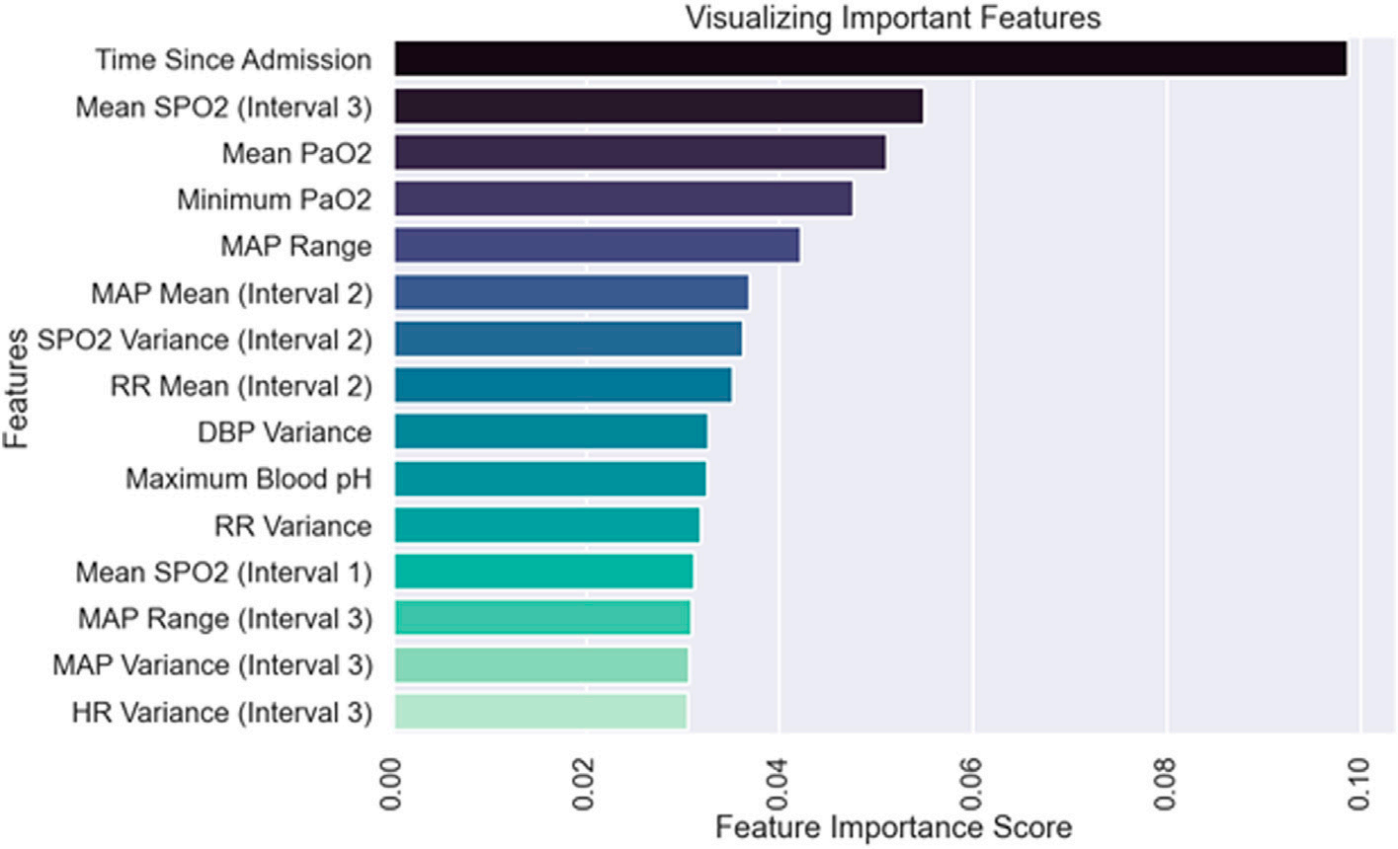
Top 15 significant Features from Cohort 1 manually derived features (M4).

**FIGURE 7:**
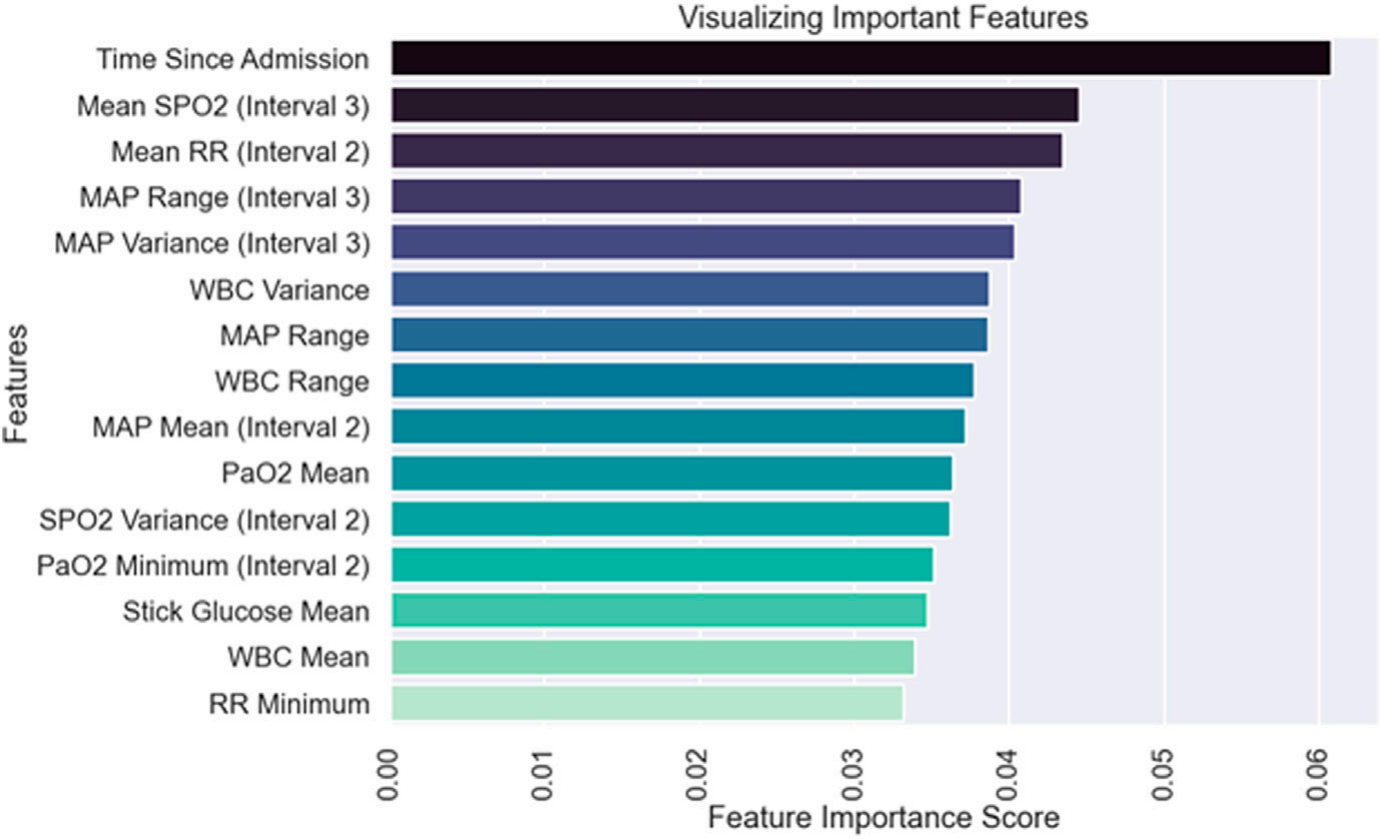
Top 15 significant Features from Cohort 2 manually derived features (M5).

The feature importance provides insight into the models. The top 15 feature importance scores for each model are plotted respectively in Figures 3–7. Time since admission was the most important feature in all models, except for M3 which used automated features from the high-frequency PTS data. While random forest variable importance does not indicate the variable’s relationship with the outcome, this is consistent with published data that VAC risk increases over time (Klompas, 2014). Model M3’s ability to provide better predictions without relying on time since admission indicates it has found physiologic features more closely associated with VAC than time since admission. The top eight features of model M3 are derived from MAP, followed by signals derived from HR, RR, DBP, and SpO_2_. In the best performing manual model, M4, mean SpO_2_ in last interval is second most important, followed by PaO_2_ statistics, then MAP statistics.

Strengths of our approach include the use of VAC as a prediction target. VAC has been demonstrated to be a clinically valid construct for patient decompensation while ventilated that is associated with worse outcomes. The feature generation and selection methodology is scalable to dense time series data. Compared to manual feature engineering, it is able to find more informative features that improve model outcome. While the automated features are not as widely recognized as standard statistical features, they are defined transformations that can be analyzed. For example, FFT and CWT coefficients correspond to specific frequencies or patterns occurring in the data. This analyzability stands in contrast to deep learning approaches where learned features are inherent to the neural network structure and cannot be easily analyzed. Finally, our use of the MIMIC cohort makes this analysis easy to reproduce and compare with other approaches.

Our model development and evaluation had several limitations. The patient cohort is from a single institution. The small number of patients with sufficient data necessitated use of cross-validation for model development and prevented evaluation of the model on a held-out dataset. The clinical origin of the dataset resulted in missingness and made some features prohibitive to obtain. During early empiric analysis of window sizes the small dataset and level of missingness may have caused overfitting for larger 48 h windows, leading to selection of 36 h model window. Our sampling strategy lead to 1:1 balance of positive and negative samples. This difference from the clinical incidence may bias metrics, lowering the AUROC and increasing AUPRC. These limitations will be addressed in future work by a) external validation of the model with a dataset from another institution and b) use of additional features obtained from ADT records, billing information, comorbidity indices such as Elixhuaser’s (Elixhauser et al., 1998).

The VAC definition has several limitations. The variables that define a VAC, while more objective than those used for the previous VAP construct (Magill et al., 2013) are under clinician control and rely on timely adjustment. Failure to wean FiO_2_/PEEP while a patient is improving could mask a subsequent VAC. Similarly, escalation of FiO_2_ and PEEP off the ARDSnet protocol (Brower et al., 2004) could trigger a VAC. One solution would be to balance these settings with a measure of oxygenation, such as PaO_2_ or SpO_2_. Finally, the VAC focuses primarily on oxygenation, and may miss decompensation that requires increased ventilation.

This work raises several questions for future work. First, given that automated feature generation outperformed manual features on the high frequency data, where does the frequency cut-off occur, and can higher frequencies further improve performance. Second, can automated features be made more interpretable.

### 4.1 Future directions

This model is still capable of improvement. Application of neural networks to raw waveform data rather than uniform down-sampling of the physiological time series data may provide greater accuracy or earlier predictions for real-time usage in the ICU. This model can also be externally validated on other critical care databases as to determine its functionality across different datasets. With such improvements, a model could be created to provide an hourly risk score of the patient’s status, or a sliding time window for clinicians to understand when a patient’s vital signs worsen, as to signal the need for appropriate clinical interventions.

## 5 Conclusion

We developed risk prediction models specific to the mechanically ventilated ICU population. The models’ ability to classify patients accurately compares favorably to the current classification standard using APACHE risk scores (Gursel and Demirtas, 2006). Though this model relies on existing records, the methodology suggests feasibility in further use of high-frequency data. Future work using more computationally intensive models and higher-frequency patient data may increase relevant classification metrics even further.

## Data Availability

The original contributions presented in the study are included in the article/Supplementary Material, further inquiries can be directed to the corresponding author.

## References

[B1] Blackburn H. N. Clark M. T. Moss T. J. Young J. S. Moorman J. R. Lake D. E. (2017). External validation in an intermediate unit of a respiratory decompensation model trained in an intensive care unit. Surgery 161 (3), 760–770. 10.1016/j.surg.2016.09.018 27894709

[B2] Brower R. G. Lanken P. N. MacIntyre N. Matthay M. A. Morris A. Ancukiewicz M. (2004). Higher versus lower positive end-expiratory pressures in patients with the acute respiratory distress syndrome. N. Engl. J. Med. 351 (4), 327–336. 15269312 10.1056/NEJMoa032193

[B3] Calvert J. S. Price D. A. Chettipally U. K. Barton C. W. Feldman M. D. Hoffman J. L. (2016). A computational approach to early sepsis detection. Comput. Biol. Med. 74, 69–73. 10.1016/j.compbiomed.2016.05.003 27208704

[B4] Christ M. Braun N. Neuffer J. Kempa-Liehr A. W. (2018). Time series FeatuRe extraction on basis of scalable hypothesis tests (tsfresh – a Python package). Neurocomputing 307, 72–77. 10.1016/j.neucom.2018.03.067

[B5] Cocoros N. M. Kleinman K. Priebe G. P. Gray J. E. Logan L. K. Larsen G. (2016). Ventilator-associated events in neonates and children—a new paradigm. Crit. Care Med. 44 (1), 14–22. 10.1097/CCM.0000000000001372 26524075 PMC10884951

[B6] Dasta J. F. McLaughlin T. P. Mody S. H. Piech C. T. (2005). Daily cost of an intensive care unit day: The contribution of mechanical ventilation. Crit. Care Med. 33 (6), 1266–1271. 10.1097/01.ccm.0000164543.14619.00 15942342

[B7] Elixhauser A. Steiner C. Harris D. R. Coffey R. M. (1998). Comorbidity measures for use with administrative data. Med. Care 36 (1), 8–27. 10.1097/00005650-199801000-00004 9431328

[B8] Fang W. F. Fang Y. T. Huang C. H. Chen Y. M. Chang Y. C. Lin C. Y. (2020). Risk factors and associated outcomes of ventilator-associated events developed in 28 days among sepsis patients admitted to intensive care unit. Sci. Rep. 10 (1), 12702. 10.1038/s41598-020-69731-3 32728165 PMC7391677

[B9] Gursel G. Demirtas S. (2006). Value of Apache II, sofa and CPIS scores in predicting prognosis in patients with ventilator-associated pneumonia. Respiration 73 (4), 503–508. 10.1159/000088708 16205047

[B10] Harrison A. M. Gajic O. Pickering B. W. Herasevich V. (2016). Development and implementation of sepsis alert systems. Clin. Chest Med. 37 (2), 219–229. 10.1016/j.ccm.2016.01.004 27229639 PMC4884325

[B11] Huber W. Findeisen M. Lahmer T. Herner A. Rasch S. Mayr U. (2020). Prediction of outcome in patients with ARDS: A prospective cohort study comparing ARDS-definitions and other ARDS-associated parameters, ratios and scores at intubation and over time. PLoS ONE 15, e0232720. 10.1371/journal.pone.0232720 32374755 PMC7202606

[B12] Johnson A. E. W. Pollard T. J. Shen L. Lehman L. wei H. Feng M. (2016). MIMIC-III, a freely accessible critical care database. Sci. Data 3 (1), 160035. 10.1038/sdata.2016.35 27219127 PMC4878278

[B13] Klompas M. (2014). Ventilator-associated conditions versus ventilator-associated pneumonia: Different by design. Curr. Infect. Dis. Rep. 16 (10), 430. 10.1007/s11908-014-0430-0 25129117

[B14] Klompas M. (2019). Ventilator-associated events: What they are and what they are not. Respir. Care 64 (8), 953–961. 10.4187/respcare.07059 31346070

[B15] Magill S. S. Klompas M. Balk R. Burns S. M. Deutschman C. S. Diekema D. (2013). Developing a new, national approach to surveillance for ventilator-associated events: Executive summary. Clin. Infect. Dis. 57 (12), 1742–1746. 10.1093/cid/cit577 24280662 PMC3840402

[B16] Moody B. George V. Mauricio C. Gari S. (2017). Ikaro. MIMIC-III waveform database [internet]. PhysioNet; 2017. [cited 2021 Apr 1]. Available from: https://physionet.org/content/mimic3wdb/1.0/.

[B17] Muscedere J. Sinuff T. Heyland D. K. Dodek P. M. Keenan S. P. Wood G. (2013). The clinical impact and preventability of ventilator-associated conditions in critically ill patients who are mechanically ventilated. Chest 144 (5), 1453–1460. 10.1378/chest.13-0853 24030318

[B18] Pedregosa F. Varoquaux G. Gramfort A. Michel V. Thirion B. Grisel O. (2011). Scikit-learn: Machine learning in Python. Mach. Learn PYTHON 6.

[B19] Rajkomar A. Dean J. Kohane I. (2019). Machine learning in medicine. N. Engl. J. Med. 380 (14), 1347–1358. 10.1056/nejmra1814259 30943338

[B20] Scherpf M. Gräßer F. Malberg H. Zaunseder S. (2019). Predicting sepsis with a recurrent neural network using the MIMIC III database. Comput. Biol. Med. 113, 103395. 10.1016/j.compbiomed.2019.103395 31480008

[B21] Schmidt M. Bailey M. Sheldrake J. Hodgson C. Aubron C. Rycus P. T. (2014). Predicting survival after extracorporeal membrane oxygenation for severe acute respiratory failure. The Respiratory Extracorporeal Membrane Oxygenation Survival Prediction (RESP) score. Am. J. Respir. Crit. Care Med. 189 (11), 1374–1382. 10.1164/rccm.201311-2023OC 24693864

[B22] Tan Y. Young M. Girish A. Hu B. Kurian Z. Greenstein J. L. (2022). “Predicting respiratory decompensation in mechanically ventilated adult ICU patients,” [Internet]. medRxiv. 10.3389/fphys.2023.1125991 PMC10140580 37123253

